# A case report of acute pancreatitis with glycogen storage disease type IA in an adult patient and review of the literature

**DOI:** 10.1097/MD.0000000000022644

**Published:** 2020-10-16

**Authors:** Jiaoyu Ai, Wenhua He, Xin Huang, Yao Wu, Yupeng Lei, Chen Yu, Kivanc Görgülü, Kalliope N. Diakopoulos, Nonghua Lu, Yin Zhu

**Affiliations:** a The Department of Gastroenterology; b The Department of Radiology, The First Affiliated Hospital of Nanchang University, Jiangxi, China; c Comprehensive Cancer Center Munich, Klinikum rechts der Isar, Technical University Munich, Munich Germany.

**Keywords:** acute pancreatitis, adult patient, clinical treatment, glycogen storage disease type IA, hypertriglyceridemia

## Abstract

**Rationale:**

Glycogen storage disease type IA (GSD IA) is an inherited disorder of glycogen metabolism characterized by fasting hypoglycemia, hyperuricemia, and hyperlipidemia including hypertriglyceridemia (HTG). Patients have a higher risk of developing acute pancreatitis (AP) because of HTG. AP is a potentially life-threatening disease with a wide spectrum severity. Nevertheless, almost no reports exist on GSD IA-induced AP in adult patients.

**Patient concerns:**

A 23-year-old male patient with GSD 1A is presented, who developed moderate severe AP due to HTG.

**Diagnoses:**

The GSD 1A genetic background of this patient was confirmed by Sanger sequencing. Laboratory tests, along with abdominal enhanced-computed tomography, were used for the diagnosis of HTG and AP.

**Interventions:**

This patient was transferred to the intensive care unit and treated by reducing HTG, suppressing gastric acid, inhibiting trypsin activity, and relieving hyperuricemia and gout.

**Outcomes:**

Fifteen days after hospital admission, the patient had no complaints about abdominal pain and distention. Follow-up of laboratory tests displayed almost normal values. Reexamination by computed tomography exhibited a reduction in peripancreatic necrotic fluid collection compared with the initial stage.

**Lessons:**

Fast and long-term reduction of triglycerides along with management of AP proved effective in relieving suffering of an adult GSD IA-patient and improving prognosis. Thus, therapeutic approaches have to be renewed and standardized to cope with all complications, especially AP, and enable a better outcome so that patients can master the disease.

## 1 Introduction

Glycogen storage disease type I (GSD I) is an extremely rare and inherited metabolic disorder occurring with an incidence of approximately 1/100,000 to 1/400,000 live births in the general Caucasian population^[1,2]^ and induced by deficiencies of the glucose-6-phosphatase (G6Pase)/glucose-6-phosphate translocase (G6PT) complex. Specifically, glycogen storage disease type IA (GSD IA) results from mutations of the gene *G6PC*, located on chromosome 17q21^[3]^ and encoding for the G6Pase catalytic subunit, which causes loss of G6Pase function and accounts for approximately 80% of GSD I.^[4]^ Defects in G6Pase are followed by accumulation of glycogen in the liver and other organs, and patients present with fasting hypoglycemia, hyperuricemia, lactic acidosis, and hyperlipidemia which is a secondary risk factor of acute pancreatitis (AP).^[5]^ Hypertriglyceridemia (HTG) is the main form of hyperlipidemia, leading to AP.^[6]^ We report an adult case of AP with GSD IA, which was induced by HTG.

## 2 Case report

A 23-year-old male patient presented with acute persistent pain in the upper abdomen accompanied by nausea and vomiting and was hospitalized in May 2019.

The detailed patient history is as follows. Diagnosis of GSD IA was established according to clinical features and genetic background (see below). At birth abdominal distention (frog belly) and hepatomegaly were detected. At 7 years of age the patient suffered twice from epistaxis with a much longer bleeding time than normal. Because of growth failure and obvious clinical symptoms, the patient had to frequently undergo hospital examinations and treatments. For GSD 1A therapy, medical recommendation included nutrient supplementation of cornstarch at 12 years of age. To reduce hyperlipidemia ezetimibe tablets 10 mg qd, fenofibrate 200 mg qd, and orlistat 0.12 g tid were prescribed at 13 years of age along with febuxostat tablets 40 mg qd for relieving hyperuricemia and gout. The patient is of short stature with a baby face due to beard growth defects while in puberty. In April 2016, this patient was admitted to the hospital for the first time due to HTG-AP. He was cured after treatment.

The GSD 1A genetic background of this patient was confirmed by testing performed at the Shanghai institute of pediatric medicine in August 2017. Sanger sequencing of the *G6PC* gene (NM_000151.3) revealed 2 distinct mutations in exon 5 of both alleles: c.648G>T (p.L216L) inherited from the father and c.986A>T (p.K329 M) inherited from the mother. Disease-associated c.648G>T mutation frequencies of G6PC are 88% in Japanese patients and 36% in Chinese patients.^[7]^ c.986A>T has not yet been reported in the literature. Thus, it might be a novel discovery of pathogenic G6PC mutation in GSD IA.

The delivery of this patient was normal and full-term. His parents were not consanguineous. This patient has 2 healthy younger siblings. He got married 2 years ago and had a healthy child last year.

**Figure 1:**
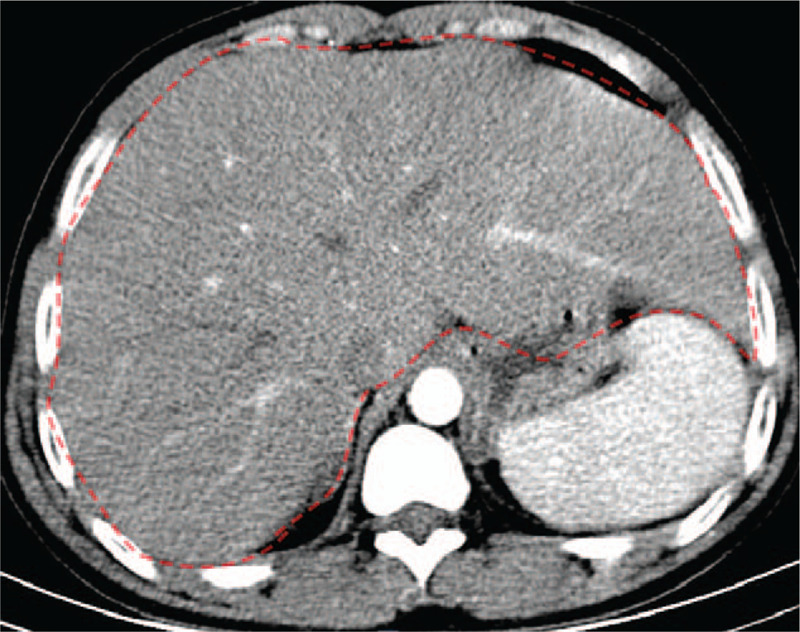
Abdominal contrast-enhanced computed tomography image displaying hepatomegaly (red area).

**Figure 2:**
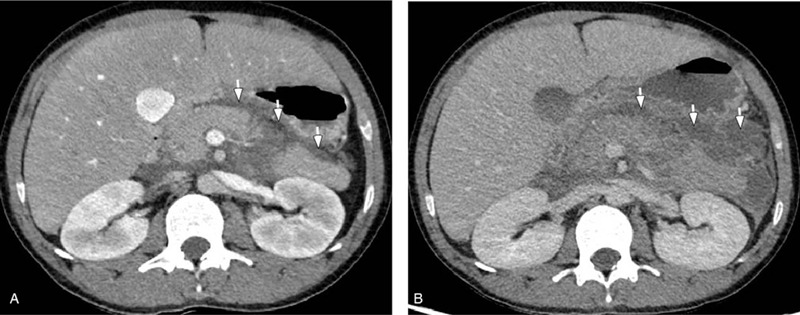
A: Abdominal contrast-enhanced CT showing edema and peripancreatic necrotic fluid collection (white arrows). B: Abdominal contrast-enhanced CT showing increased peripancreatic necrotic fluid collection (white arrows). CT = computed tomography.

Directly after hospitalization in May 2019, laboratory tests displayed blood biochemical results as follows: amylase 209 U/L (reference values 35–135), lipase 475 U/L (reference values 0–60), triglyceride 49.64 mmol/L (reference values 0–1.7) (=4393.14 mg/dL), total cholesterol 15.51 mmol/L (reference values 0–5.7) (=599.8 mg/dL), glucose 5.82 mmol/L (reference values 3.9–6.1) as well as uric acid 662 umol/L (reference values 208–428). Urine routine examination provided a value of 1.0 g/L urinary protein (reference value 0). Arterial blood gas analysis revealed respiratory failure (PO2/FiO2 =144 mm/Hg).^[8]^ Abdominal computed tomography (CT) exhibited:

1. Liver: Hepatomegaly (Fig. 1);
2. Pancreas: AP with edema in the pancreas and peripancreatic necrotic fluid collection (Fig. 2A);
3. Left renal calyces: multiple small stones.

Integrating the disease history of this patient, 3 diagnoses were established:

1. moderately severe HTG-AP;
2. GSD IA;
3. hyperuricemia.

This patient was treated as follows: fasting within 24 hours and resuscitation with intravenous infusion in the initial period; pantoprazole 40 mg q12h for gastric acid suppression; somatostatin 3 mg q12h and ulinastatin 10 WU qd for inhibition of trypsin activity; combination of enoxaparin sodium 4000 IU q12 h with insulin for initial 3 days followed by fenofibrate 200 mg qd for decreasing HTG; ezetimibe 10 mg qd and orlistat 0.12 g tid for reduction of hyperlipemia; febuxostat tablets 40 mg qd and sodium bicarbonate tablets 1000 mg q8h for relieving hyperuricemia and gout; lactulose oral solution 30 ml tid, Chinese rhubarb 100 ml tid and artificially assisted purgative enema for promoting peristalsis. In the meantime, butorphanol tartrate by micro pump was used for pain relief. One day later, enteral nutrition was given by nutritional canal. After 5 days, compound Azintamide Enteric-coated tablets (2 tablets tid) were used for digestive enzyme supplementation and live triple Combined capsule of Bifidobacterium, Lactobacillus, and Enterococcus (1 tablet bid) was used to restore the intestinal flora.

**Figure 3:**
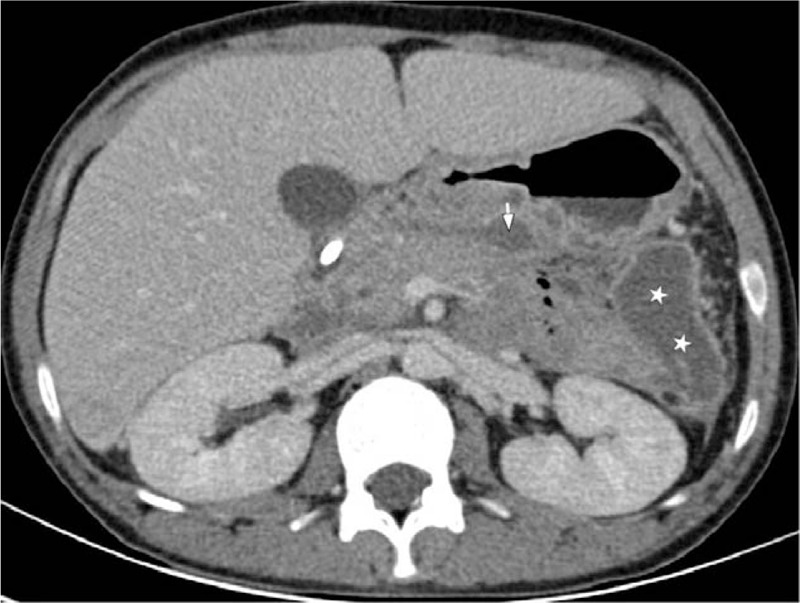
Abdominal contrast-enhanced CT displaying decreased peripancreatic necrotic fluid collection compared with Figure 2B (white arrow) and defined inflammatory wall (white stars).

Five days after hospitalization, reexamination by abdominal CT displayed an increase in peripancreatic necrotic fluid collection compared with the initial stage image (Fig. 2B vs Fig. 2A). However, after an additional 10 days, the patient had no complaints about abdominal pain and distention. Follow-up of laboratory tests displayed blood biochemical result as follows: amylase 102 U/L, triglyceride 9.01 mmol/L (796.95 mg/dL), total cholesterol 6.13 mmol/L (237.2 mg/dL), glucose 2.3 mmol/L, and uric acid 232 umol/L. Arterial blood gas analysis revealed normalized oxygenation index of PO2/FiO2. Repetition of abdominal enhanced-CT exhibited a reduction in peripancreatic necrotic fluid collection compared with the previous images with a defined inflammatory wall (Fig. 3 vs Fig. 2B). Following recovery, this patient was discharged. Until now, around 8 months after leaving the hospital, the patient maintains normal health by means of regularly taking ezetimibe,fenofibrate, and orlistat.

## 3 Discussion

GSD IA is the most common of the glycogen storage diseases,^[4]^ mainly caused by abnormal glycogen storage and hypoglycemia when fasting due to dysfunctional glycogen metabolism. Without adequate metabolic treatment, patients with GSD IA die during infancy or childhood after overwhelming hypoglycemia and acidosis. Those who survive are stunted in physical growth and delayed in puberty because of chronically low insulin levels. Therefore, the key treatment is to maintain normal blood sugar levels and inhibit various metabolic disorders secondary to hypoglycemia, thereby relieving clinical symptoms.

Up to date, specific approaches for this disease are lacking and dietary adjustment is the cornerstone of treatment. Uncooked cornstarch is a widely used treatment method for GSD IA.^[9–11]^ After preparation, due to the large molecular weight, cornstarch is slowly digested and stays in the intestine for longer time. It gradually releases glucose to maintain blood sugar at a normal level. This patient reported here started to utilize cornstarch as dietary treatment with frequent meals (every 6 hours) when he was 12-years old, crucially enhancing his life expectancy. Cornstarch also conduces to delaying the complications fatal to GSD IA patients, as for example HTG, hyperuricemia, gout, kidney stones, liver adenoma, etc (Table 1). Nonetheless, new treatments have to be established to manage GSD IA complications since dietary adjustments cannot prevent them.

**Table 1:**
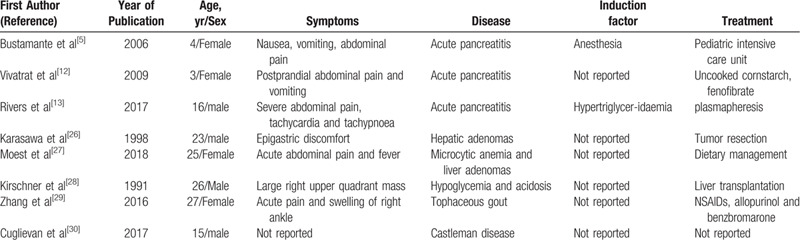
Previously published case reports of diseases induced by GSD IA.

The present report is the first one describing an adult patient suffering from GSD 1A-induced AP. Up to date, only few articles exist describing the cases of GSD IA-induced AP^[5,12,13]^; however, not including adult patients thus highlighting the importance of this report.

HTG is a well-known cause of AP, accounting for approximately 10% to 19% of all cases.^[14,15]^ Hyperlipidemia can be separated into hypercholesterolemia and HTG. In contrast to HTG, hypercholesterolemia does not cause AP.^[6]^ In China, HTG-mediated AP is the second leading cause of AP accounting for around 14% of all cases but has a higher mortality rate than that of the biliary/gallstone-induced AP which is the primary leading cause of AP.^[16]^ The most important risk factor are elevated triglycerides, reaching up to 11.3 mmol/L (1000 mg/dL).^[17]^ Accordingly, the presented patient had a very high TG level of 49.64 mmol/L (4393.14 mg/dL). However, the pathogenesis of HTG-AP is poorly understood. Free fatty acids are reported as one of the key factors to initiate the pathogenesis of HTG-AP.^[18]^ Hydrolysis of accumulating triglycerides by the enzyme lipase in the pancreatic capillaries triggers free fatty acids release resulting in activation of trypsinogen and damages to pancreatic capillaries by free radicals.^[19,20]^

The patient reported here was treated with fluid resuscitation and enteral nutrition in the early phase, as recommended by the guidelines for AP.^[21]^ More importantly, the combination of heparin and insulin was used for the initial phase of AP to reduce serum triglycerides. Although high-volume hemofiltration was effective in rapidly reducing HTG, one randomized controlled trial from He et al compared high-volume hemofiltration with the combination of low-molecular-weight heparin and insulin, showing no difference in terms of clinical outcomes and costs.^[22]^ For long-term management of hyperlipidemia/HTG, cholesterol absorption inhibitor and fibrates were utilized. As described previously, the present patient had AP 3 years ago and was cured. One main cause of AP-recurrence was the pausing of the cholesterol absorption inhibitor ezetimibe and fibrates fenofibrate, 20 days before recurrence. Therefore, and in line with the publication reported by Guo et al,^[18]^ it is very important to reduce triglycerides in order to prevent AP.

The reported patient was also suffering from hyperuricemia, which is a common complication of GSD IA. Hyperuricemia occurs due to accumulation of the G6PC-substrate glucose 6-phosphate. Elevated glucose 6-phosphate is subsequently metabolized by the pentose phosphate pathway and leads to the excess production of purine. Purine catabolism then results into uric acid. Simultaneously, excretion of uric acid through renal tubules is reduced due to competitive inhibition by organic acids,^[23]^ collectively contributing to increased levels of blood uric acid. Hyperuricemia is usually accompanied by gout during puberty. It can be improved in GSD IA-patients by controlling whole body metabolism. However, persistent hyperuricemia mostly results in gout attacks. Xanthine oxidase inhibitors (allopurinol or febuxostat) are the first-line medicine for reducing uric acid. The patient presented herein currently takes febuxostat tablets to control gout caused by hyperuricemia showing promising results. The complications of hyperuricemia commonly include renal calcification and kidney stones.^[11]^ The CT result of this patient indicated multiple small stones in renal calyces, most probably caused by elevated uric acid.

The patient herein exhibited most of the known GSD IA-complications. GSD IA causes not only great pain and physical injury but also shortens life expectancy of patients. Hospitalization costs are high and treatments focus only on preventing GSD IA-complications. Therefore, it is urgent to find new ways for an effective cure of the disease.

Gene therapy is expected to be one possible cure for GSD IA.^[7,24]^ Koeberl et al injected an adeno-associated viral vector (AAV2/8) carrying G6PCcDNA into 2-week old G6PC^−/−^ mice. 14 days after injection, the liver started to shrink and fasting blood glucose level gradually increased. The median survival extended for 7 months.^[25]^ Nevertheless, gene therapy is still in the exploratory stage. There are many problems to be solved relating to long-term safety and effectiveness. In addition, research projects focusing on GSD I are rare. However, gene therapy may show promising results in the future.

## 4 Conclusion

This is the first report of HTG-AP in an adult-GSD IA patient. The combination-treatment of heparin and insulin is efficient for reducing triglycerides in a short time, and the combination of cholesterol absorption inhibitor and fibrates is stably decreasing HTG / hyperlipidemia over a long term. Moreover, AP as the complication of GSD IA can be effectively managed by comprehensive treatment, thereby relieving suffering of GSD IA-patients and improving prognosis. Thus, standard therapeutic approaches have to be established to manage the complications and improve therapy of this rare genetic disease.

## Acknowledgment

We would like to thank the patient's family for agreeing on publishing the data. The patient has provided informed consent for publication of this case report.

## Author contributions

Jiaoyu Ai provided clinical diagnosis and care and drafted the manuscript. Wenhua He, Xin Huang, Yao Wu, Yupeng Lei provided clinical diagnosis and care. Chen Yu provided clinical diagnosis. Kivanc Görgülü and Kalliope N.Diakopoulos provided extensive discussion and edited the manuscript. Nonghua Lu and Yin Zhu provided clinical care and designed the manuscript.
